# Leaf epidermal micromorphology in *Aspidistra* (Asparagaceae): diversity and taxonomic significance

**DOI:** 10.3897/phytokeys.185.72259

**Published:** 2021-11-15

**Authors:** Nikolay A. Vislobokov, Long-Fei Fu, Yi-Gang Wei, Maxim S. Nuraliev

**Affiliations:** 1 Department of Higher Plants, Faculty of Biology, M.V. Lomonosov Moscow State University, Moscow 119234, Russia M.V. Lomonosov Moscow State University Moscow Russia; 2 Joint Russian-Vietnamese Tropical Scientific and Technological Center, Cau Giay, Hanoi, Vietnam Joint Russian-Vietnamese Tropical Scientific and Technological Center Hanoi Vietnam; 3 Guangxi Key Laboratory of Plant Conservation and Restoration Ecology in Karst Terrain, Guangxi Institute of Botany, Guangxi Zhuang Autonomous Region and Chinese Academy of Sciences, Guilin, China Guangxi Institute of Botany, Guangxi Zhuang Autonomous Region and Chinese Academy of Sciences Guilin China

**Keywords:** *Aspidistra*, epidermis, identification key, papillae, SEM

## Abstract

Micromorphological characters of leaf epidermis were investigated in 69 species of *Aspidistra* using scanning electron microscopy. Sculpture of epidermis varies from smooth to verrucose and rugose in the genus. The abaxial epidermis of some species bears papillae, whereas the adaxial surface uniformly lacks the papillae. Sculpture type of epidermis and density of papillae are generally found to be stable characters at a species level. The infraspecific variation of epidermis sculpture, where present, ranges from smooth to verrucose or from verrucose to rugose. Micromorphological characters of leaf epidermis are shown to have potential taxonomic significance in *Aspidistra*; in combination with the type of shoot structure, they allow to subdivide the species into 13 groups. The groups are largely incongruent with floral morphological traits. An identification key to the studied species of *Aspidistra* based on vegetative characters (gross leaf and shoot morphology and characters of leaf epidermis) is presented.

## Introduction

*Aspidistra* Ker Gawl., belonging to the family Asparagaceae, is a large genus of herbaceous plants which inhabits tropical and subtropical forests of Asia. In our estimate, the genus comprises about 200 species. *Aspidistra* is remarkable for its extremely diverse flower morphology (Tillich 2005, 2008, 2014; Averyanov and Tillich 2012; Tillich et al. 2017; Tillich and Averyanov 2018). Floral characters are most important in taxonomy of *Aspidistra*, whereas vegetative characters are rarely used for species identification: most of the species have similar habit and are hardly distinguishable without flowers. The majority of representatives of *Aspidistra* are characterized by creeping rhizome without aerial shoots. Only a few species have erect stem (e.g. *A.erecta* Yan Liu & C.I Peng and *A.globosa* Vislobokov & Nuraliev), representing a group easily recognizable by vegetative morphology (Vislobokov et al. 2016). Another non-floral character that was shown to have taxonomic significance in *Aspidistra* is the distribution of foliage leaves along the shoot (De Wilde and Vogel 2006; Averyanov and Tillich 2014). Shoots of *Aspidistra* consist of repeatedly developing elementary shoots, each elementary shoot bearing several cataphylls followed by one to several foliage leaves (Vislobokov et al. 2014, 2017). The species of *Aspidistra* can be divided into two groups: the first group is characterized by solitary leaves (i.e., one foliage leaf per elementary shoot), and in the second group the leaves are arranged in tufts (i.e., 3–5 foliage leaves per elementary shoot). Additionally, these groups of species differ in the gross morphology of leaf. In most species of the first group, the leaf is divided into petiole and blade (e.g. *A.arnautovii* Tillich, *A.formosa* (Tillich) Aver. & Tillich, *A.subrotata* Y.Wan & C.C.Huang), with blade of various shape. By contrast, all species of the second group have narrowly elliptic or linear leaves lacking a petiole but gradually tapering towards base (e.g. *A.carnosa* Tillich, *A.hainanensis* W.Y.Chun & F.C.How, *A.viridiflora* Vislobokov & Nuraliev). The second group is much smaller with respect to species number than the first one.

Despite the usefulness of the vegetative characters of *Aspidistra* outlined above, they are far from being enough for identification to the species level, because numerous species often share the same combination of these characters. Thus, the precise identification of *Aspidistra* in non-flowering condition in most cases is impossible at the current state of knowledge. At the same time, identification of sterile plants of *Aspidistra* appears to be highly demandable due to several features of reproductive biology of this genus. The flowers in *Aspidistra* are usually developed at the ground level, often hidden by leaf litter, and their search requires special efforts (Tillich 2005). The field recognition of species of *Aspidistra* is also complicated by their common sympatric occurrence: three or four (and up to six) species are often recorded in a given forest, where they sometimes grow side by side forming mixed populations (Nuraliev et al. 2017). Moreover, the flowering takes place only in a particular season, which differs among the species, so that only a part of individuals of the genus (if any) are usually observed to produce flowers in a given forest. A widely used technique of specimen identification in *Aspidistra* is collecting sterile living material and obtaining the flowers under cultivation (Averyanov and Tillich 2014, 2015; Vislobokov et al. 2019b). However, this method requires a preliminary estimation of the number of the species inhabiting a given area.

Micromorphological characters, including those of vegetative organs, sometimes appear sufficiently diverse to serve as a useful instrument for taxonomy and species identification. This approach has already been successfully applied for *Dracaena* Vand. ex L., another genus of Asparagaceae (Klimko et al. 2018), as well as for certain genera of Caryophyllaceae, Lamiaceae and Myrtaceae (Haron and Moore 1996; Mostafavi et al. 2013; Krawczyk and Głowacka 2015). To date, micromorphology has never been investigated in *Aspidistra*. In the present study, we investigated micromorphological characters of leaf epidermis in this genus. Our goals were (1) to evaluate diversity of adaxial and abaxial leaf epidermis in *Aspidistra* including cell sculpture and density of papillae; (2) to determine infraspecific variation of these characters; (3) to analyze taxonomic significance of characters of leaf epidermis by delineating species groups on the basis of these characters; (4) to compile an identification key to the studied species of *Aspidistra* based on vegetative features, including leaf and shoot morphology and the characters of leaf epidermis.

## Materials and methods

### Specimens

Fully developed foliage leaves were collected from living plants of *Aspidistra* found in nature (during fieldwork in China and Vietnam) as well as cultivated in the Botanical Garden Munich-Nymphenburg (BGMN), the Botanical Institute of the Russian Academy of Sciences (BIN), the Main Botanical Garden of the Russian Academy of Sciences (MBG) and the Singapore Botanic Gardens (SBG) (Appendix 1). The leaves were fixed and stored in 70% ethanol. All the studied specimens possessed floral material, and identification of the plants used in this study was verified by investigation of floral structure. In total, 113 specimens representing 69 species of *Aspidistra* were involved in the study. Of them, 45 specimens represent type material (including types and paratypes). Each species was represented by 1 to 8 specimens, and a total of 22 species (ca. 32%) were represented by two or more specimens.

### Scanning electron microscopy (SEM)

For SEM, a single fragment ca. 5×5 mm was cut out from the leaf blade by a razor blade for each specimen. The fragment was taken from the central part of the leaf blade (equidistant from petiole/leaf base and leaf apex), equidistantly from midvein and leaf margin, between secondary veins. The dissected material was transferred from 70% ethanol to 100% acetone via 80% and 96% ethanol followed by an 1: 1 mixture of ethanol (96%) and acetone (100%). The material was critical-point dried using an HCP-2 critical point dryer (Hitachi, Japan). Dried samples were divided in two equal parts by a razor blade, which then were mounted onto stubs with different sides exposed, using double-sided sticky tape. The mounted specimens were coated with gold using an Eiko IB-3 ion-coater (Eiko Engineering, Japan) and observed using a CamScan 4DV (CamScan, UK) scanning electron microscope at Moscow State University.

## Morphological traits

The following traits of leaf epidermis were investigated: (1) size and shape of epidermal cells; (2) fine relief of the outer periclinal cell wall: micro-sculpture of epidermis surface; and (3) curvature of outer periclinal wall: presence and density of papillae on epidermis. Size of epidermal cells and density of stomata were measured once for each specimen. Density of papillae was measured by counting number of papillae within a frame 100×100 µm in at least two repeats in each specimen. Standard terminology of surface sculpturing patterns mainly follows Barthlott (1981).

The identification key was compiled on the basis of original data on leaf micromorphology and data on gross vegetative morphology available from Liang and Tamura (2000), Li (2004) and the species protologues.

## Results

### Diversity of micromorphological traits

Leaf epidermis of examined species of *Aspidistra* consists of elongated tetragonal cells 60–160 µm long and 10–40 µm wide with straight boundaries. The leaves are amphistomatic. Stomata are of anomocytic type. Density of stomata is 50–170 per 1 mm^2^ on abaxial surface, and very low on adaxial side (less than 10 per 1 mm^2^). Guard cells are 25–40 µm long and 5–10 µm wide.

**Figure 1.**
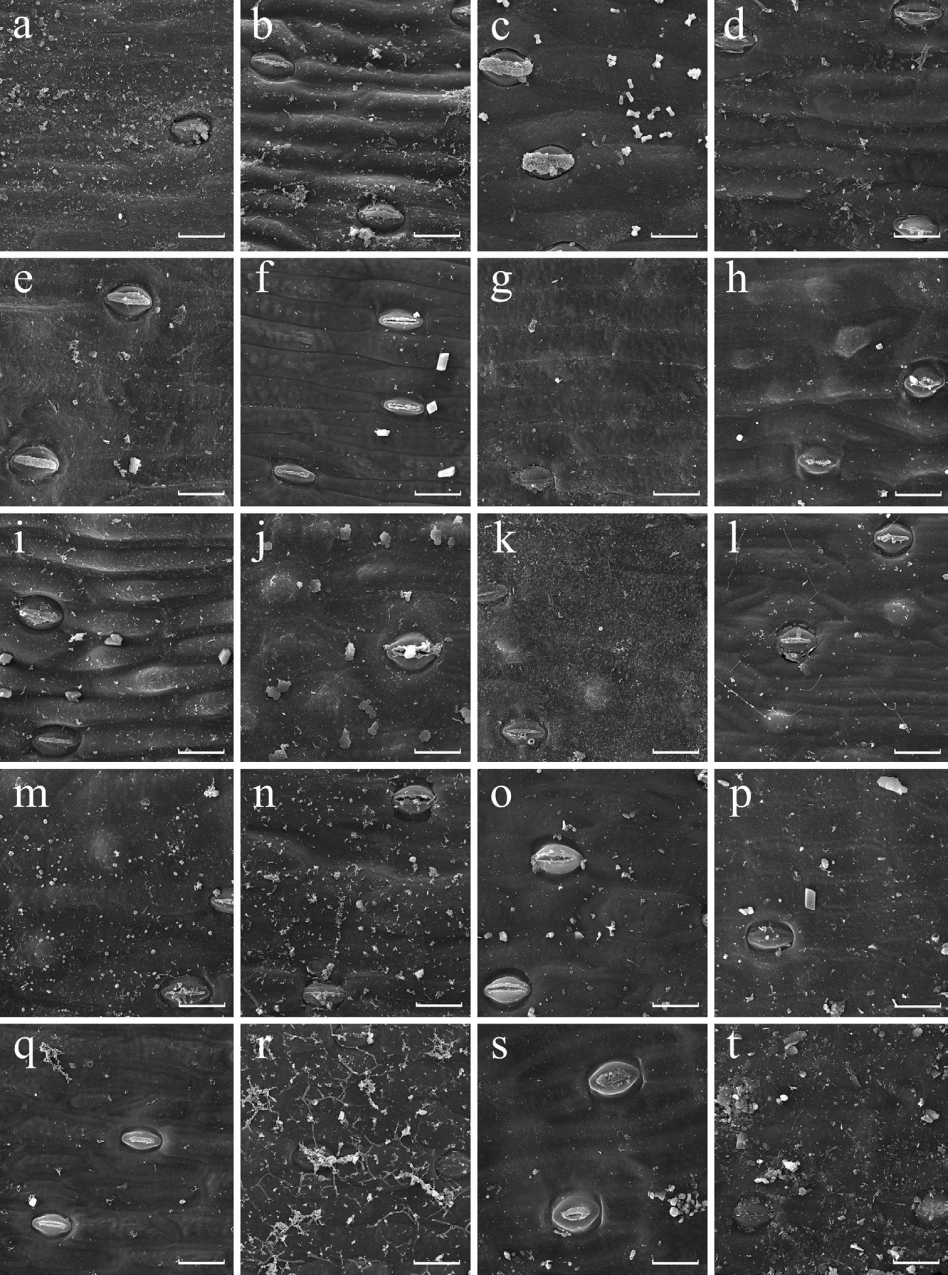
SEM images of abaxial leaf epidermis of *Aspidistra*; morphological groups I (a–g), II (h–m) and III (n–t) (partly). **a***A.graminifolia***b***A.hainanensis* (s.n.) **c***A.linearifolia***d***A.oviflora* (13394) **e***A.triradiata***f***A.viridiflora***g***A.yingjiangensis***h***A.carnosa***i***A.cylindrica***j***A.hainanensis* (11/1394) **k***A.longifolia***l***A.oviflora* (2018.14340.01) **m***A.larutensis***n***A.atroviolacea***o***A.clausa***p***A.claviformis***q***A.dolichanthera* (2016.12354.01) **r***A.erecta***s***A.jingxiensis***t***A.lurida*. Scale bars: 30 µm.

**Figure 2.**
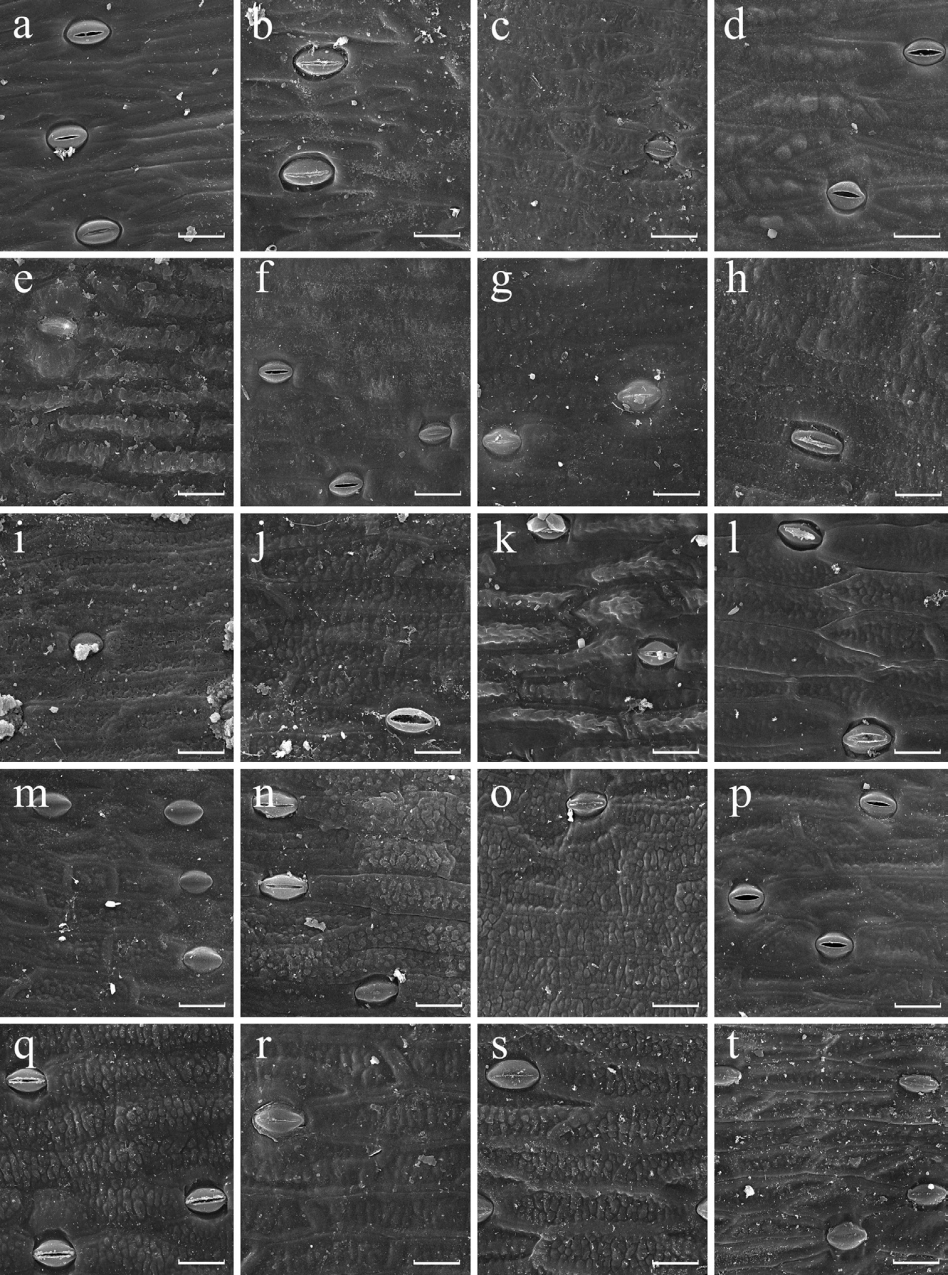
SEM images of abaxial leaf epidermis of *Aspidistra*; morphological groups III (a–c) (partly) and IV (d–t). **a***A.petiolata***b***A.renatae***c***A.sessiliflora***d***A.basalis***e***A.lateralis***f***A.medusa***g***A.typica***h***A.connata* (V/0490) **i***A.bella***j***A.erosa* (JLS2972) **k***A.globosa***l***A.gracilis***m***A.laotica* (20122018) **n***A.mirostigma***o***A.phanluongii* (2015.11347.01) **p***A.sarcantha* (JLS2962) **q***A.subrotata* (2015.11350.01) **r***A.sutepensis***s***A.truongii* (2013/2461) **t***A.vietnamensis*. Scale bars: 30 µm.

**Figure 3.**
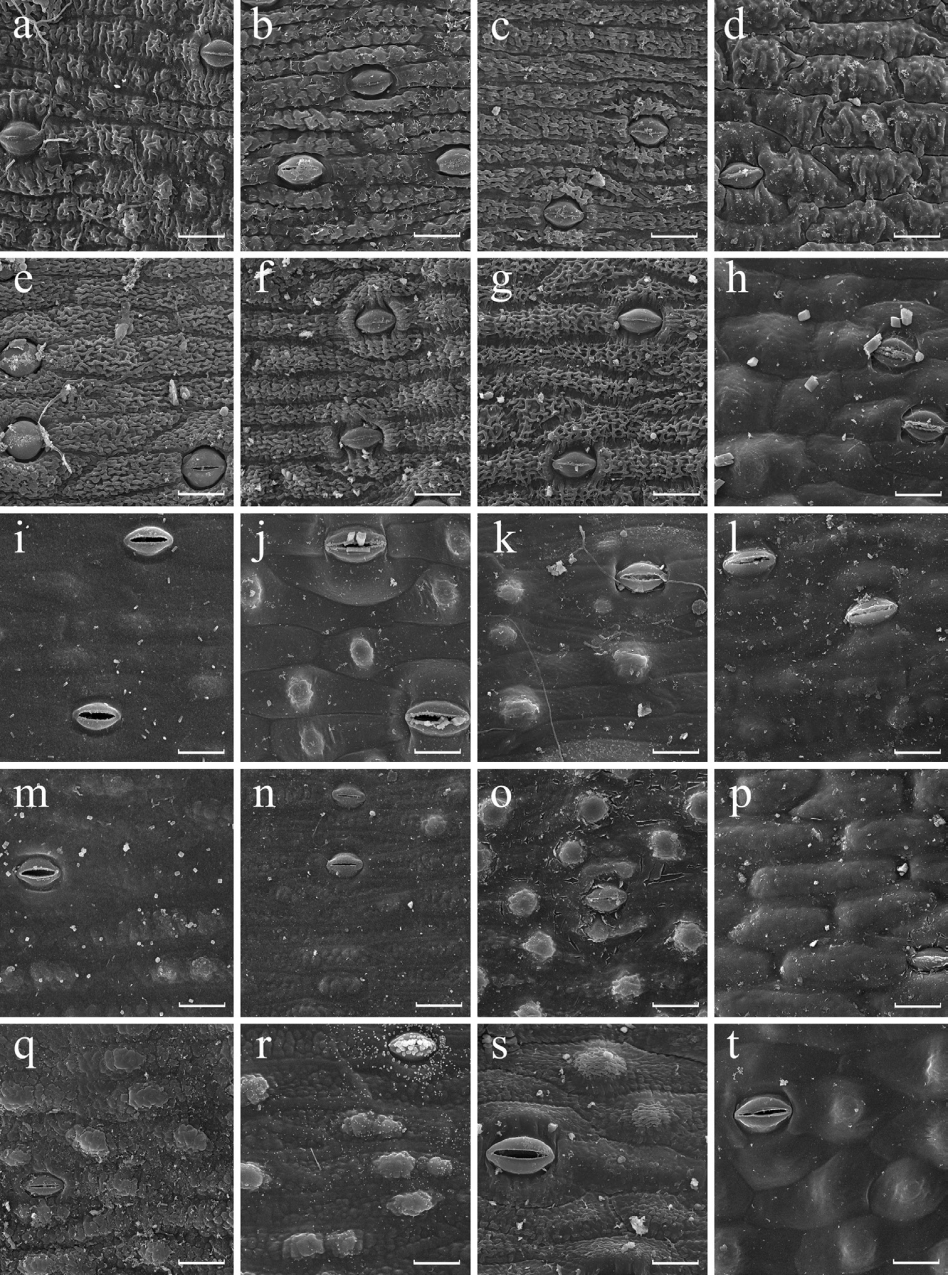
SEM images of abaxial leaf epidermis of *Aspidistra*; morphological groups V (a–g), VI (h–l, o), VII (m, n, p–r), VIII (s) and IX (t) (partly). **a***A.erosa* (JLS2906) **b***A.locii* (86-169) **c***A.lubae***d***A.xuansonensis***e***A.corniculata***f***A.foliosa* (97/2360) **g***A.multiflora***h***A.fungilliformis* (2016.12350.02) **i***A.geastrum***j***A.longipetala***k***A.papillata***l***A.tillichiana***m***A.connata* (97/2358) **n***A.semiaperta***o***A.bicolor***p***A.clausa***q***A.opaca* (18035) **r***A.subrotata* (A.s.5) **s***A.minor***t***A.hekouensis*. Scale bars: 30 µm.

**Figure 4.**
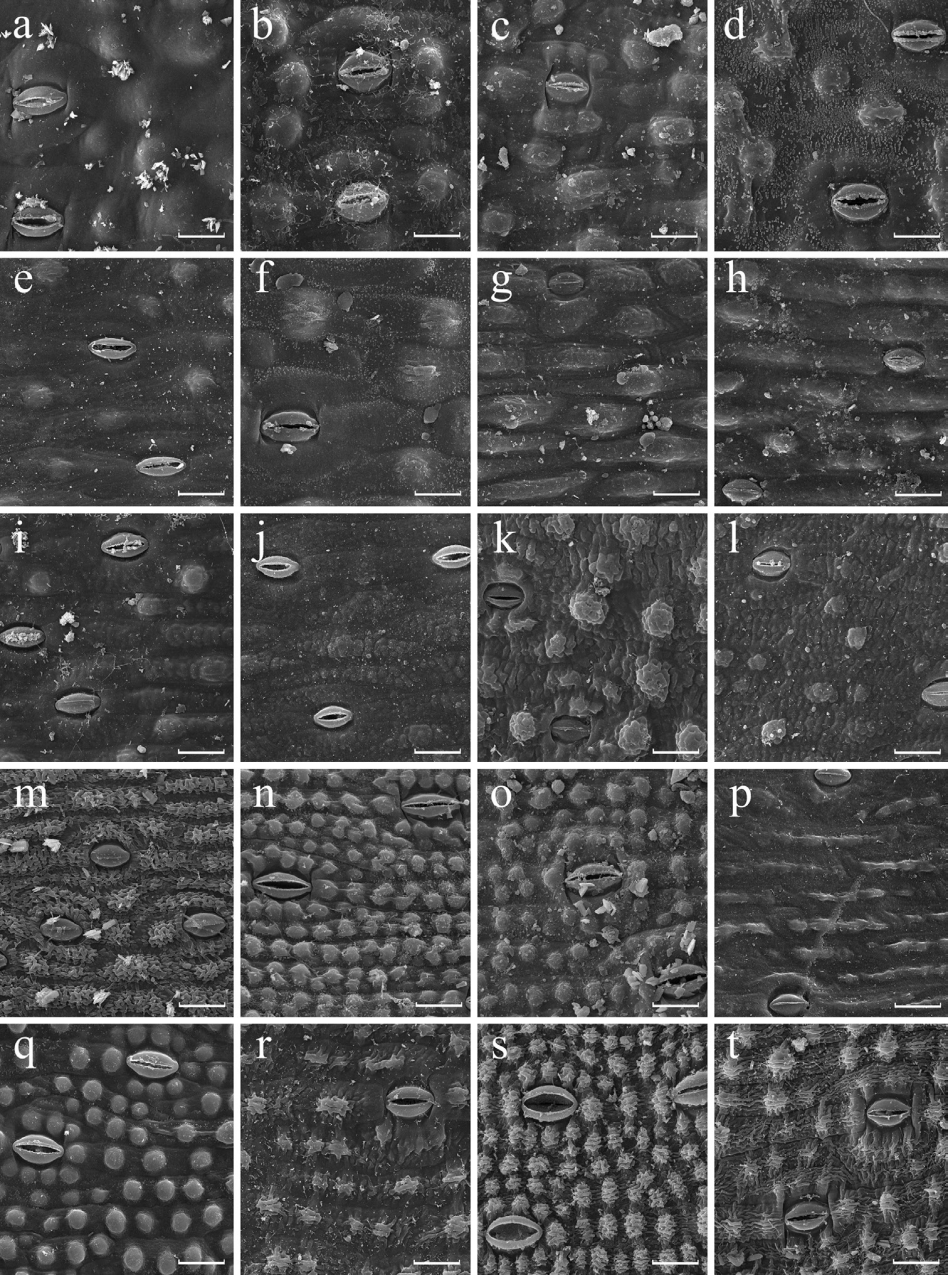
SEM images of abaxial leaf epidermis of *Aspidistra* morphological groups IX (a–f) (partly), X (g–l), XI (m), XII (n–q) and XIII (r–t). **a***A.longanensis***b***A.lutea* (96/3126) **c***A.sinensis***d***A.stricta***e***A.subrotata* (20121895) **f***A.superba***g***A.arnautovii* (18566) **h***A.connata* (96/3119) **i***A.nutans***j***A.subrotata* (JLS2989) **k***A.opaca* (2013/2460W) **l***A.subrotata* (04/1769) **m***A.zinaidae***n***A.bogneri* (9311) **o***A.grandiflora***p***A.letreae***q***A.magnifica***r***A.formosa* (2015.11376.01) **s***A.jiewhoei* (JLS1218) **t***A.marasmioides* (2015.11354.01). Scale bars: 30 µm.

Epidermis surface is smooth or sculptured to various degrees (Figs 1–4). Within the observed variation, we recognize two types of epidermis sculpture: *verrucose* and *rugose*. Verrucose surface bears rough irregularities, short wide ridges or projections (e.g. Figs 2e, j, k, o, 3q, r). Rugose surface has numerous fine narrow tortuous folds (e.g. Figs 3e-g, 4m).

Epidermis of about a half of the studied specimens is smooth on both surfaces. In the other specimens, the adaxial and abaxial epidermis is either uniformly or differently micro-ornamented (or one of the surfaces is smooth). Usually the sculpture is pronounced to a greater extent on the abaxial surface than on the adaxial one. Verrucose sculpture, if present, is usually found on both sides, or only abaxially with smooth adaxial epidermis. Rugose epidermis is usually expressed on both leaf sides or rarely only on the abaxial side, with the adaxial side being verrucose. Thus, only the abaxial epidermis is illustrated (Figs 1–4).

Adaxial epidermis of foliage leaves of all studied specimens is uniformly epapillate. Abaxial epidermis of about a half of the studied specimens bears papillae (e.g. Figs 3o, 4n, s). Papillae are hemispherical, 10–30 µm in diameter, with one to several of them per epidermal cell. Density of papillae varies from 1 to 45.5 papillae per 0.01 mm^2^. We recognize three categories of papillae density: low, 1–3.5 papillae per 0.01 mm^2^; medium, 4–7.5 papillae per 0.01 mm^2^; high, 8–45.5 papillae per 0.01 mm^2^.

Additionally, we have found that shape and density of papillae on the secondary veins (not employed in the main part of our study) is different in some specimens from those of the epidermis located between the secondary veins.

The main results of investigation of each species (epidermis sculpture and the presence and density of papillae) are presented in Appendix 1.

### Infraspecific variation

Size and shape of epidermal cells of investigated species does not reveal any species-specific pattern: their infraspecific variation is nearly as broad as interspecific variation.

The micro-sculpture of abaxial and adaxial epidermis is, in contrast, generally constant (fixed) at a species level. These traits show stability in at least 16 out of 22 species of *Aspidistra* represented by two or more specimens in the present study. We found that only in several species the sculpture of at least one leaf side is variable; it varies either between smooth and verrucose, or between verrucose and rugose. We have not observed any species with variation between smooth and rugose sculpture. For example, in two investigated specimens of *A.arnautovii* adaxial epidermis is verrucose, whereas in the other three specimens (Fig. 4g) the epidermis is smooth (Appendix 1, group X). Epidermis sculpture of *A.formosa* also varies from verrucose to rugose at adaxial and abaxial surface (Fig. 4r). Finally, two studied specimens of *A.erosa* Aver., Tillich, T.A.Le & K.S.Nguyen have verrucose adaxial surface but differ in having rugose vs. verrucose surface of abaxial epidermis (Figs 2j, 3a).

Presence and density of papillae is also stable in 16 out of 22 species of *Aspidistra* represented by two or more specimens. Density of papillae on abaxial epidermis varies only slightly within some species. Appreciable variation was found in *A.arnautovii* (4–7 papillae per 0.01 mm^2^; Fig. 4g), *A.bogneri* (38–45.5 papillae per 0.01 mm^2^; Fig. 4n), *A.lutea* Tillich (4–6 papillae per 0.01 mm^2^; Fig. 4b) and *A.opaca* Tillich (4–5.5 papillae per 0.01 mm^2^; Figs 3q, 4k); nevertheless, each of these species fits a single category of papillae density (low, medium or high) proposed above. The most significant variation of this feature was found in *A.connata* Tillich (0–5 papillae per 0.01 mm^2^), *A.hainanensis* (0–1.5 papillae per 0.01 mm^2^; Figs 1b, j), *A.oviflora* Aver. & Tillich (0–2.5 papillae per 0.01 mm^2^; Fig. 1d, l) and *A.subrotata* (0–6 papillae per 0.01 mm^2^; Fig. 2q, 3r, 4e); they are categorized as showing variation from absence of papillae to low or medium density of papillae.

## Discussion

### Combinations of vegetative characters found in *Aspidistra*

We built a space of logical possibilities for all the studied specimens with regard to the following characters of vegetative morphology: type of shoot, sculpture of adaxial and abaxial epidermis, and density (and presence) of papillae. The specimens showed 23 combinations of these traits. Considering infraspecific variation of some traits, we combined the studied specimens of *Aspidistra* into 13 groups (Appendix 1) in order to make specimens of the same species belong to one group. This method allowed categorizing specimens of 62 species, whereas 7 species (*A.clausa* Vislobokov, *A.connata*, *A.erosa*, *A.hainanensis*, *A.opaca*, *A.oviflora*, *A.subrotata*) showed too high variation of traits so that specimens of each species got into several groups. The brief description of these groups is presented in Table 1.

**Table 1.** Groups of the examined specimens of *Aspidistra* recognized here, and traits on which the groups are based.

**Group**	**Type of shoot (leaves solitary/tufted)**	**Sculpture of adaxial epidermis**	**Sculpture of abaxial epidermis**	**Density of papillae on abaxial epidermis**
I	tufted	smooth/verrucose	smooth/verrucose	no papillae
II	tufted	smooth	smooth/verrucose	low
III	solitary	smooth/verrucose	smooth	no papillae
IV	solitary	smooth/verrucose	verrucose	no papillae
V	solitary	verrucose/rugose	rugose	no papillae
VI	solitary	smooth/verrucose	smooth	low
VII	solitary	smooth/verrucose	verrucose	low
VIII	solitary	rugose	rugose	low
IX	solitary	smooth	smooth	medium
X	solitary	smooth/verrucose	verrucose	medium
XI	solitary	rugose	rugose	medium
XII	solitary	smooth	smooth	high
XIII	solitary	verrucose/rugose	verrucose/rugose	high

Some species possess a unique combination of traits, e.g. *Aspidistraminor* Vislobokov, Nuraliev & M.S.Romanov (Fig. 3s, group VIII) is readily distinguishable from all other studied species by leaf epidermis finely rugose on both sides bearing abaxially papillae of low density. *Aspidistrazinaidae* Aver. & Tillich (Fig. 4m, group XI) likewise possesses a unique set of traits, which is similar to that of *A.minor* and differs in medium density of papillae.

We recognized no correlation between the morphological groups of species outlined above and geographical distribution of the species.

The availability of recognition of the morphological groups indicates that the characters under study show an infraspecific variation that is narrow enough, and the interspecific diversity that is broad enough to be applied to taxonomy for most of the studied species of *Aspidistra*. In other words, the characters possess a taxonomic signal. The identification key provided below is a reflection of this conclusion.

### Correlations between leaf micromorphology and floral structure

Most of the groups of species outlined here on the basis of vegetative characters do not show any correlation with floral traits. We were able to recognize only several cases of such correlation, which are addressed below.

*Aspidistramirostigma* Tillich & Škorničk., *A.phanluongii* Vislobokov and *A.sarcantha* Aver., Tillich, T.A.Le & K.S.Nguyen are similar in floral groundplan and shape: they share trimerous flowers with urceolate perigone, short style and wide stigma with its margin adjoined to the wall of perigone tube (Vislobokov et al. 2013; Leong-Škorničková et al. 2014; Averyanov et al. 2019). These species are demonstrated here to share epapillate leaf epidermis with verrucose sculpture at adaxial and abaxial surface, all belonging to group IV (Fig. 2n, o, p). The micromorphological features are thus in concordance with floral features in this group of species. At the same time, group IV comprises 13 more species, and the floral diversity of the entire group is remarkable high.

*Aspidistraatroviolacea* Tillich and *A.renatae* Bräuchler (treated as A.atroviolaceavar.renatae (Bräuchler) Tillich & Aver. by Tillich and Averyanov 2018) are similar in having dark violet campanulate perigone and mushroom-shaped pistil (i.e. with slender style and hemispherical stigma) (Bräuchler and Ngoc 2005; Tillich 2005). Both species have epapillate smooth leaf epidermis (Figs 1n, 2b; belonging to group III), again in concordance with the floral features.

A group of five species, *A.corniculata* Vislobokov, *A.erosa*, *A.foliosa* Tillich, *A.lubae* Aver. & Tillich and *A.multiflora* Aver. & Tillich, shares trimerous flowers with mostly reddish-purple campanulate perigone and mostly white mushroom-shaped pistil (Tillich 2005; Averyanov and Tillich 2014, 2015; Averyanov et al. 2019; Vislobokov et al. 2019a). This group is uniformly characterized by finely rugose surface of abaxial leaf epidermis and absence of papillae (Figs 3a, c, e, f, g; group V). On the other hand, *A.locii* Arnautov & Bogner (Fig. 3b, group V) and *A.xuansonensis* Vislobokov (Fig. 3d; group V) have the same micromorphological traits, but possess distinctly different flowers.

### Taxonomically uncertain groups of species in *Aspidistra* in the light of micromorphological data

The general stability of the micromorphological characters at the species level demonstrated here in *Aspidistra* allows to discuss taxonomy of complicated groups of species with employment of the newly obtained data.

Representatives of *Aspidistra* with tufted leaves (i.e., with several foliage leaves per elementary shoot) were considered to form a group of closely related species (De Wilde and Vogel 2006; Tillich and Averyanov 2012). Various authors proposed different taxonomic decisions to accommodate the diversity of plants with this morphology. The entire group was regarded as a single variable species *A.longifolia* Hook.f. s.l. by Phonsena and De Wilde (2010). Tillich and Averyanov (2012), in contrast, outlined the SE Asian part of this group as *A.hainanensis* species complex comprising several species (and excluded *A.longifolia* s.str. decribed from Assam, India from this complex). Here we follow the latter viewpoint, as it describes better the floral variation of these plants. Within our study, the specimens of *Aspidistra* with tufted leaves form two morphological groups (group I and II) which differ from each other in the presence of papillae on leaf epidermis (absence vs. presence with low density). The leaf epidermis of all these species is usually smooth but slightly tuberous in some cases. Although both characters vary within *A.hainanensis* species complex, we consider the variation not to be very significant for species delimitation, because it does not exceed the range of infraspecific variation found in some other species (e.g. *A.arnautovii* and *A.subrotata*). Thus, our data do not contradict the idea of phylogenetic closeness of these species.

Several other taxa with uncertain boundaries form a group here referred to as *A.subrotata* species complex. It includes *A.subrotata* with several described infraspecific taxa and *A.connata* with two proposed varieties. *Aspidistraconnata* was recently suggested to be treated as a synonym of *A.subrotata*, as the absence of any considerable differences in their floral structure was shown during investigation of extensive material (Averyanov et al. 2018). *Aspidistrasubrotata* is one of the most widely distributed species of the genus: it was originally described from China (Wan and Huang 1987), and subsequently reported from numerous localities in Vietnam (Tillich 2005, 2014), Thailand (Phonsena and De Wilde 2010) and Laos (Averyanov and Tillich 2017). *Aspidistraconnata* is also known from China (Xu et al. 2010) and Vietnam (Tillich 2005; Leong-Škorničková et al. 2014). Both species inhabit diverse habitats and show extremely high diversity in size of flowers and leaves, shape of the leaf blade, and shape and coloration of the stigma (Averyanov and Tillich 2017). In the present study, *A.subrotata* and *A.connata* are expediently treated as distinct species in order to compare their micromorphological characters. Eight specimens of *A.subrotata* and four specimens of *A.connata* were investigated. We demonstrate that both species show very high diversity of leaf micromorphology. In both species, sculpture of epidermis varies between smooth and verrucose (but never rugose) adaxially as well as abaxially, and the abaxial side varies from being completely epapillate to having medium density of papillae. Accordingly, specimens of each species fall into several morphological groups (*A.subrotata* – IV, VI, VIII, IX; *A.connata* – IV, VI, IX). Thus, features of leaf micromorphology do not provide any clues for delimitation of *A.subrotata* complex; on the other hand, they do not contradict the idea of distinctness of *A.subrotata* and *A.connata*, because the variation found in each species is higher than the variation found in most other species of *Aspidistra*.

### Key for identification of the studied species of *Aspidistra* based on epidermis micromorphology and gross vegetative morphology

**Table:** 

1	Leaves grouped on shoot by 3–5 (3–5 foliage leaves per elementary shoot), sessile; blade narrowly elliptic to linear, 1–5 cm wide, 15–50 times as long as wide	**2**
–	Leaves solitary (one leaf per elementary shoot), petiolate or sessile; blade of various shape	**6**
2	Adaxial epidermis epapillate; abaxial epidermis with sparse papillae (papillae density low: 1–3.5 papillae per 0.01 mm^2^)	**3**
–	Adaxial and abaxial epidermis epapillate	**4**
3	Epidermis smooth adaxially, verrucose abaxially	***A.larutensis***
–	Epidermis smooth on both sides	***A.carnosa***, ***A.hainanensis***, ***A.longifolia***, ***A.oviflora***
4(2)	Adaxial and abaxial epidermis smooth	***A.graminifolia***, ***A.hainanensis***, ***A.linearifolia***, ***A.oviflora***, ***A.triradiata***
–	Epidermis verrucose at least on one side	**5**
5	Epidermis smooth adaxially, verrucose abaxially	***A.viridiflora***
–	Epidermis verrucose on both sides	***A.yingjiangensis***
6(1)	Adaxial and abaxial epidermis smooth and epapillate; leaf petiolate	**7**
–	Epidermis sculptured (verrucose or rugose) or papillate at least on one side; leaf petiolate or sessile	**9**
7	Plant with aerial erect to ascending stem ca. 50 cm high	***A.erecta***
–	Plant without aerial stem	**8**
8	Blade narrowly lanceolate to narrowly elliptic, 1.5–4.5 cm wide	***A.atroviolacea***, ***A.clausa***, ***A.renatae***
–	Blade ovate to elliptic, 5–15 cm wide	***A.claviformis***, ***A.dolichanthera***, ***A.jingxiensi*s**, ***A.lurida***, ***A.petiolata***, ***A.sessiliflora***
9(6)	Adaxial and abaxial epidermis epapillate	**10**
–	Adaxial epidermis epapillate, abaxial epidermis papillate	**23**
10	Epidermis verrucose at least on one side (and never rugose)	**11**
–	Epidermis finely rugose at least on one side	**18**
11	Plant with erect stem	**12**
–	Plant without erect stem	**14**
12	Aerial stem up to 50 cm high	***A.globosa***
–	Aerial stem 3–20 cm high	**13**
13	Aerial stem 3–5 cm high, blade 8–16 cm long	***A.laotica***
–	Aerial stem ca. 20 cm high, blade 20–25 cm long	***A.lateralis***
14(11)	Blade narrowly elliptic to narrowly lanceolate, 8–20 times as long as wide	**15**
–	Blade elliptic to ovate, 2–7 times as long as wide	**16**
15	Petiole 3–5 cm long	***A.basalis***
–	Petiole 15–32 cm long	***A.erosa***, ***A.gracilis***, **A.subrotatavar.angustifolia**
16(14)	Epidermis verrucose on both sides	***A.bella***, ***A.mirostigma***, ***A.phanluongii***, ***A.sarcantha***, ***A.subrotata***, ***A.sutepensis***, ***A.truongii***, ***A.vietnamensis***
–	Epidermis verrucose on one side, smooth on the other side	**17**
17	Petiole longer than blade	***A.medusa***
–	Petiole equal to or shorter than blade	***A.connata***, ***A.typica***
18(10)	Epidermis finely rugose on both sides	**19**
–	Epidermis verrucose adaxially, finely rugose abaxially	**20**
19	Blade 1.5–3.7 cm wide	***A.corniculata***, ***A.foliosa***
–	Blade 4–10 cm wide	***A.multiflora***
20(18)	Blade elliptic, 5–11 cm wide	***A.locii***, ***A.xuansonensis***
–	Blade lanceolate, 1.5–5 cm wide	**21**
21	Blade equal to or insignificantly longer than petiole	***A.erosa***
–	Blade 2–10 times as long as petiole	**22**
22	Blade 12–20 cm long, 4–6 times as long as wide	**A.lubaevar.lubae**
–	Blade 20–35 cm long, 8–13 times as long as wide	**A.lubaevar.lancifolia**
23(9)	Density of papillae on abaxial epidermis low (1–3.5 papillae per 0.01 mm^2)^	**24**
–	Density of papillae on abaxial epidermis medium to high (4–45.5 papillae per 0.01 mm^2^)	**26**
24	Epidermis finely rugose on both sides	***A.minor***
–	Epidermis smooth or verrucose on both sides	**25**
25	Epidermis smooth on both sides	***A.fungilliformis***, ***A.geastrum***, ***A.longipetala***, ***A.papillata***, **A.tillichianavar.latifolia**
–	Epidermis verrucose at least on one side	***A.bicolor***, ***A.clausa***, ***A.connata***, ***A.opaca***, ***A.semiaperta***, ***A.subrotata***
26(23)	Density of papillae on abaxial epidermis medium (4–7.5 papillae per 0.01 mm^2^)	**27**
–	Density of papillae on abaxial epidermis high (8–45.5 papillae per 0.01 mm^2^)	**30**
27	Epidermis finely rugose on both sides	***A.zinaidae***
–	Epidermis smooth or verrucose on both sides	**28**
28	Epidermis verrucose at least on one side	***A.arnautovii***, ***A.connata***, ***A.nutans***, ***A.opaca***, ***A.subrotata***
–	Epidermis smooth on both sides	**29**
29	Petiole 2–5 cm long, blade 3–7 times as long as petiole	***A.lutea***, ***A.sinensis***
–	Petiole 9–28 cm long, blade equal to or insignificantly longer than petiole	***A.hekouensis***, ***A.longanensis***, ***A.stricta***, ***A.subrotata***, ***A.superba***
30(26)	Epidermis finely rugose (rarely verrucose) on both sides	**31**
–	Epidermis smooth on both sides	**33**
31	Rhizome with very short internodes (foliage leaves crowded); blade 20–30 × 8.5–13 cm	***A.jiewhoei***
–	Rhizome with long internodes (foliage leaves spaced 8–25 mm apart); blade 10–17 × 5–8 cm	**32**
32	Petiole 10–15 cm long, blade 10–12 × 5 cm; epidermis finely rugose on both sides	***A.marasmioides***
–	Petiole 15–20 cm long, blade 14–17 × 7–8 cm; epidermis finely rugose or verrucose at least on one side	***A.formosa***
33(30)	Blade narrowly lanceolate, 1.5–3 cm wide, 23–30 times as long as wide	***A.letreae***
–	Blade lanceolate, elliptic or ovate, 5–14 cm wide, 6–9 times as long as wide	**34**
34	Petiole absent or inconspicuous	***A.bogneri***
–	Petiole distinctly present, usually 30 cm long or longer	***A.grandiflora, A.magnifica***

## Conclusions

Micromorphological characters of leaf epidermis show sufficiently high diversity in the genus *Aspidistra*, and relatively low infraspecific variation in most of its species. The following variable characters are recognized: sculpture of adaxial and abaxial epidermis (smooth, verrucose and rugose) and the presence and density of papillae at abaxial side of leaf (absent, with low, medium and high density). Combined with characters of gross vegetative morphology, they allow recognition of 13 basic types of vegetative morphology in *Aspidistra*. We constructed an identification key for species of *Aspidistra* in sterile condition on the basis of the newly obtained micromorphological data and earlier known macromorphological traits. The key allows to identify a species to a group containing one to eight species. The results demonstrate considerable taxonomic significance of micromorphological features in *Aspidistra*.

## Appendix 1

**Table A1.** Species and specimens of *Aspidistra* examined, and traits of vegetative morphology. The species are arranged according to their belonging to the morphological groups, and in the alphabetical order within the groups. Note that some species are represented in more than one group.

Species	Living specimens studied		Herbarium voucher	Country of origin	Type of shoot (leaves solitary / tufted)	Sculpture of adaxial epidermis	Sculpture of abaxial epidermis	Density of papillae distribution on abaxial epidermis**	Group
	Greenhouse hosting specimen*	Garden accession number; field collector’s number (if differs from that of herbarium voucher)							
*A.graminifolia*	BIN	1285	*Averyanov AL 84* (isotype, MW: MW0751740)	Vietnam	tufted	smooth	smooth	no papillae	I
*A.hainanensis*	BIN	s.n.	-	unknown	tufted	smooth	smooth	no papillae	
*A.linearifolia*	BGMN	2011/0980; *Joschko s.n.*	-	unknown	tufted	smooth	smooth	no papillae	
*A.oviflora*	MBG	2013.12433.01	*Vislobokov 13062* (MW: MW0735044)	Vietnam	tufted	smooth	smooth	no papillae	
*A.oviflora*	BIN	13394; *Maisak TM1052*	*Averyanov et al. CPC5425* (isotype, LE: LE01050400)	Vietnam	tufted	smooth	smooth	no papillae	
*A.triradiata*	MBG	2016.12342.01	*Vislobokov et al. G30* (MW: MW0753785)	China	tufted	smooth	smooth	no papillae	
*A.viridiflora*	MBG	2015.11381.01	*Nuraliev et al. 1280* (holotype, MW: MW0754789)	Vietnam	tufted	smooth	verrucose	no papillae	
*A.yingjiangensis*	BIN	251964	-	unknown	tufted	verrucose	verrucose	no papillae	
*A.carnosa*	BGMN	96/3122W; *Arnautov 96-102*	*Tillich 4476* (holotype, M: M0213536)	Vietnam	tufted	smooth	smooth	low	II
*A.cylindrica*	MBG	2015.11382.01	*Kuznetsov et al. 1357* (holotype, MW: MW0595646)	Vietnam	tufted	smooth	smooth	low	
*A.hainanensis*	BGMN	11/1394	*Tillich 5717* (M: MSB159621)	unknown	tufted	smooth	smooth	low	
*A.longifolia*	BGMN	15/1593	-	unknown	tufted	smooth	smooth	low	
*A.oviflora*	MBG	2018.14340.01	-	unknown	tufted	smooth	smooth	low	
	BIN	14386	*Averyanov et al. CPC7491* (LE: LE01050072)	Vietnam	tufted	smooth	smooth	low	
*A.larutensis*	BGMN	s.n.	-	unknown	tufted	smooth	verrucose	low	
*A.atroviolacea*	BGMN	97/2363	*Bogner 2309* (paratype, M: M0213530)	Vietnam	solitary	smooth	smooth	no papillae	III
*A.clausa*	MBG	2014.12431.01	*Vislobokov 14097* (holotype, MW: MW0595637)	Vietnam	solitary	smooth	smooth	no papillae	
*A.claviformis*	MBG	2016.12351.01	*Vislobokov et al. G71* (MW: MW0753784)	China	solitary	smooth	smooth	no papillae	
*A.connata*	BGMN	V/0490	-	unknown	solitary	verrucose	smooth	no papillae	
*A.dolichanthera*	MBG	2016.12354.01	*Vislobokov et al. G74* (MW: MW0753743)	China	solitary	smooth	smooth	no papillae	
*A.dolichanthera*	BIN	s.n.	*Averyanov CBL1675* (LE: LE01054609)	Vietnam	solitary	smooth	smooth	no papillae	III
*A.erecta*	MBG	2016.12349.01	*Vislobokov et al. G61* (MW: MW0753774)	China	solitary	smooth	smooth	no papillae	
*A.jingxiensis*	MBG	2016.12348.01	*Vislobokov et al. G51* (MW: MW0753783)	China	solitary	smooth	smooth	no papillae	
*A.lurida*	BIN	2225	*s.coll., s.n.* (LE: LE01050063)	unknown	solitary	smooth	smooth	no papillae	
*A.petiolata*	SBG	20121925; *Leong-Škorničková JLS1622*	-	Vietnam	solitary	smooth	smooth	no papillae	
*A.renatae*	BGMN	04/1772	*Brauchler et al. 3000* (holotype, M: M0243611)	Vietnam	solitary	smooth	smooth	no papillae	
*A.sessiliflora*	BIN	14448	*Averyanov AL271* (holotype, LE: LE01050440)	China	solitary	smooth	smooth	no papillae	
*A.basalis*	BGMN	2011/1397	*Tillich 5720* (holotype, M: MSB159619)	China	solitary	smooth	verrucose	no papillae	IV
*A.bella*	MBG	2019.14670	*Averyanov et al. CPC7484* (paratype, LE: LE01042157)	Vietnam	solitary	verrucose	verrucose	no papillae	
*A.erosa*	SBG	*Leong-Škorničková JLS2972*	-	unknown	solitary	verrucose	verrucose	no papillae	
*A.globosa*	from nature	-	*Kuznetsov et al. 1444* (paratype, MW: MW0595640)	Vietnam	solitary	verrucose	verrucose	no papillae	
*A.gracilis*	BGMN	2011/1395	*Tillich 5718* (holotype, M: MSB159618)	China	solitary	verrucose	verrucose	no papillae	
*A.laotica*	SBG	20122018	*Leong-Škorničková et al. JLS1802* (SING: 0192256)	Laos	solitary	verrucose	verrucose	no papillae	
	BIN	1311(1313)	*Averyanov LA-VN554* (holotype, LE: LE01032172)	Laos	solitary	verrucose	verrucose	no papillae	
*A.lateralis*	BGMN	97/2382; *Bogner 2492*	*Tillich 4361* (holotype, M: MSB004977)	Vietnam	solitary	smooth	verrucose	no papillae	
*A.medusa*	SBG	*Leong-Škorničková JLS3096*	-	Laos	solitary	smooth	verrucose	no papillae	
*A.mirostigma*	SBG	*Leong-Škorničková JLS1571*	*Leong-Škorničková et al. JLS-1571* (holotype SING)	Vietnam	solitary	verrucose	verrucose	no papillae	
*A.phanluongii*	MBG	2015.11347.01	*Nuraliev 874* (MW: MW0735057)	Vietnam	solitary	verrucose	verrucose	no papillae	
	from nature	-	*Vislobokov M0211* (paratype, MW: MW0591735)	Vietnam	solitary	verrucose	verrucose	no papillae	
*A.sarcantha*	SBG	*Leong-Škorničková JLS2914*	-	Vietnam	solitary	verrucose	verrucose	no papillae	
	SBG	JLS2962	*Leong-Škorničková et al. JLS2962* (SING: 0240186)	Vietnam	solitary	verrucose	verrucose	no papillae	
A.subrotatavar.subrotata	BGMN	97/2376 II	*Tillich 4461* (M: M0213561)	Vietnam	solitary	verrucose	verrucose	no papillae	
	MBG	2015.11350.01	*Vislobokov 13067* (MW: MW0735071)	Vietnam	solitary	verrucose	verrucose	no papillae	IV
A.subrotatavar.angustifolia	MBG	2015.11365.01	*Vislobokov 13097* (MW: MW0735079)	Vietnam	solitary	verrucose	verrucose	no papillae	IV
*A.sutepensis*	BGMN	05/2338	*Tillich 5081* (M: MSB126218)	Thailand	solitary	verrucose	verrucose	no papillae	
*A.truongii*	BGMN	2013/2461	*Leong-Škorničková et al. HB17* (paratype, SING: 0188968)	Vietnam	solitary	verrucose	verrucose	no papillae	
*A.truongii*	BGMN	2013/2461W	*Rybkova et al. HB17 6484* (M: M0210863)	Vietnam	solitary	verrucose	verrucose	no papillae	
	SBG	*Leong-Škorničková JLS1047*	-	Vietnam	solitary	verrucose	verrucose	no papillae	
*A.typica*	MBG	2017.12835.06	*Tillich 5996* (M)	Vietnam	solitary	smooth	verrucose	no papillae	
*A.vietnamensis*	BIN	786	*Averyanov et al. HAL12116a* (holotype, LE: LE01050347)	Vietnam	solitary	verrucose	verrucose	no papillae	
*A.corniculata*	from nature		*Vislobokov 18089* (isotype, MW: MW0595715)	Vietnam	solitary	rugose	rugose	no papillae	V
*A.erosa*	SBG	*Leong-Škorničková JLS2906*	-	Vietnam	solitary	verrucose	rugose	no papillae	
*A.foliosa*	BGMN	97/2360; *Bogner 2502*	*Tillich 4471* (holotype, M: M0213541)	Vietnam	solitary	rugose	rugose	no papillae	
	BGMN	97/2353	*Bogner 2803* (M)	Vietnam	solitary	rugose	rugose	no papillae	
*A.locii*	BGMN	96/3121; *Arnautov 86-112*	*Tillich 4359* (M: MSB004976)	Vietnam	solitary	verrucose	rugose	no papillae	
	BIN	86-169	*Arnautov 86-112* (LE: LE01055472)	Vietnam	solitary	verrucose	rugose	no papillae	
A.lubaevar.lancifolia	BIN	CPC1566	*Averyanov et al. CPC1566* (holotype, LE: LE01050389)	Vietnam	solitary	verrucose	rugose	no papillae	
A.lubaevar.lubae	BIN	13266	*Averyanov et al. CPC6962* (LE: LE01049991)	Vietnam	solitary	verrucose	rugose	no papillae	
*A.multiflora*	MBG	2015.11375.01	*Vislobokov 14045* (MW: MW0750152)	Vietnam	solitary	rugose	rugose	no papillae	
*A.xuansonensis*	MBG	2015.11366.01	*Vislobokov 13102* (paratype, MW: MW0591740)	Vietnam	solitary	verrucose	rugose	no papillae	
*A.bicolor*	BGMN	97/2352; Bogner 2503	*Tillich 4462* (holotype, M: M0213531)	unknown	solitary	verrucose	smooth	low	VI
*A.fungilliformis*	MBG	2016.12350.02	*Vislobokov et al. G63* (MW: MW0753782)	China	solitary	smooth	smooth	low	
	MBG	2016.12355.01	*Vislobokov et al. G82* (MW: MW0753780)	China	solitary	smooth	smooth	low	
*A.geastrum*	BGMN	97/2375G	*Tillich 4598* (holotype, M: M0213544)	Vietnam	solitary	smooth	smooth	low	
*A.longipetala*	MBG	2017.13459.01	*Vislobokov et al. G111* (MW: MW0754777)	China	solitary	smooth	smooth	low	
*A.papillata*	from nature		*Vislobokov 18087* (MW: MW0756241)	Vietnam	solitary	smooth	smooth	low	
A.tillichianavar.latifolia	BIN	13270	*Averyanov et al. CPC6841* (holotype, LE: LE01050358)	Vietnam	solitary	smooth	smooth	low	VI
*A.clausa*	from nature		*Vislobokov 18090* (MW: MW0756227)	Vietnam	solitary	verrucose	verrucose	low	VII
*A.connata*	BGMN	97/2358	*Bogner 2495* (M)	Vietnam	solitary	smooth	verrucose	low	
*A.opaca*	from nature		*Vislobokov 18035* (MW: MW0756235)	Vietnam	solitary	verrucose	verrucose	low	
*A.semiaperta*	BIN	11614	*Averyanov et al. CPC1566b* (LE: isotype, LE01050394)	Vietnam	solitary	smooth	verrucose	low	
A.subrotatavar.subrotata	from nature		*Vislobokov et al. A.s.5* (MW: MW0735059)	Thailand	solitary	verrucose	verrucose	low	
A.subrotatavar.angustifolia	MBG	2015.11355.01	*Vislobokov et al. A.s.1* (MW: MW0735069)	Thailand	solitary	verrucose	verrucose	low	
*A.minor*	MBG	2018.14282.01	*Nuraliev et al. 1966A* (holotype, MW: MW0595716)	Vietnam	solitary	rugose	rugose	low	VIII
*A.hekouensis*	BGMN	95/2832; *Bogner 2216*	*Tillich 5005* (M: M0213547)	China	solitary	smooth	smooth	medium	IX
*A.longanensis*	BIN	260301	-	Vietnam	solitary	smooth	smooth	medium	
*A.lutea*	BGMN	96/3126; *Arnautov 76-140*	*Tillich 4481* (holotype, M: M0213551)	Vietnam	solitary	smooth	smooth	medium	
	BIN	17304	*Tillich 5003 Arnautov 76-140* (paratype, LE: LE01049990)	Vietnam	solitary	smooth	smooth	medium	
*A.sinensis*	BIN	13659	*Arnautova s.n.* (holotype, LE: LE01050480)	China	solitary	smooth	smooth	medium	
*A.stricta*	BGMN	96/3124; *Arnautov 88-110*	*Tillich 4367* (holotype, M: M0213560)	Vietnam	solitary	smooth	smooth	medium	
A.subrotatavar.subrotata	SBG	20121895; *Leong-Skornickova JLS1558*	-	Vietnam	solitary	smooth	smooth	medium	
*A.superba*	BGMN	97/2369T; *Bogner 2346*	*Tillich 4480* (paratype, M: M0213562)	Vietnam	solitary	smooth	smooth	medium	
A.arnautoviivar.arnautovii	BIN	18566	*Averyanov AL466ED4* (LE: LE01048630)	Vietnam	solitary	smooth	verrucose	medium	X
	BGMN	96/3123; *Arnautov 88-115*	*Tillich 4474* (holotype, M: M0213528)	Vietnam	solitary	smooth	verrucose	medium	
	MBG	2015.11353.01	*Vislobokov 13042* (MW: MW0735025)	Vietnam	solitary	smooth	verrucose	medium	
A.arnautoviivar.catbaensis	BGMN	96/3125; *Arnautov 88-144*	*Tillich 4460* (M: M0213526)	Vietnam	solitary	verrucose	verrucose	medium	
	MBG	2015.11352.01	*Vislobokov 13040* (MW: MW0735030)	Vietnam	solitary	verrucose	verrucose	medium	
*A.connata*	BGMN	96/3119; *Arnautov 85-722*	*Tillich 4470* (holotype, M: M0213538)	Vietnam	solitary	smooth	verrucose	medium	
	BIN	*Arnautov 85-722*	*Tillich 5486* (paratype, LE: LE01050022)	Vietnam	solitary	smooth	verrucose	medium	
*A.nutans*	BIN	13281	*Averyanov et al. CPC7158b* (holotype, LE: LE01032173)	Vietnam	solitary	smooth	verrucose	medium	
A.opacavar.opaca	BGMN	2013/2460W; *Rybkova et al. 180*	-	Vietnam	solitary	verrucose	verrucose	medium	
	BGMN	97/2359; *Bogner 2491*	*Tillich 4468* (holotype, M: M0213554)	Vietnam	solitary	verrucose	verrucose	medium	
A.opacavar.opaca	SBG	20111766	JLS1121	Vietnam	solitary	verrucose	verrucose	medium	X
A.opacavar.rugosa	BGMN	13/2460; *Rybkova et al. 180*	-	Vietnam	solitary	verrucose	verrucose	medium	
A.subrotatavar.subrotata	SBG	*Leong-Škorničková JLS2989*	-	Vietnam	solitary	smooth	verrucose	medium	
A.subrotatavar.subrotata	BGMN	04/1769	-	Vietnam	solitary	verrucose	verrucose	medium	
*A.zinaidae*	BIN	7242	*Averyanov et al. HAL11111b* (holotype, LE: LE01050435)	Vietnam	solitary	rugose	rugose	medium	XI
*A.bogneri*	BIN	9311	-	Vietnam	solitary	smooth	smooth	high	XII
	BGMN	97/2374W	*Bogner 2500* (paratype, M: M0213534)	Vietnam	solitary	smooth	smooth	high	
	BGMN	97/2404	*Bogner 2805* (paratype, M: M0213535)	Vietnam	solitary	smooth	smooth	high	
	SBG	20120086; *Leong-Škorničková JLS1436*	-	Vietnam	solitary	smooth	smooth	high	
*A.grandiflora*	BIN	11590	*Harder et al. DKH 8123* (holotype, LE: LE01054814)	Vietnam	solitary	smooth	smooth	high	
*A.letreae*	SBG	*Leong-Škorničková JLS2977*	-	Vietnam	solitary	smooth	smooth	high	
*A.magnifica*	MBG	2016.12423.01; *Nuraliev 1672*	*Romanov et al. s.n.* (MW)	Vietnam	solitary	smooth	smooth	high	
*A.formosa*	SBG	20120060; *Leong-Škorničková JLS1384*	-	Vietnam	solitary	verrucose	verrucose	high	XIII
	BGMN	1997/2367	*Tillich 5280* (holotype, M: M0213543)	unknown	solitary	verrucose	verrucose	high	
	SBG	*Leong-Škorničková JLS3178*	-	Vietnam	solitary	verrucose	verrucose	high	
	MBG	2015.11376.01	*Vislobokov 14062* (MW: MW0750154)	Vietnam	solitary	verrucose	rugose	high	
	BIN	s.n.	*Averyanov et al. CPC6412* (LE: LE01050398)	unknown	solitary	rugose	rugose	high	
*A.jiewhoei*	SBG	*Leong-Škorničková JLS1218*		Vietnam	solitary	rugose	rugose	high	
	SBG	20122069	*Leong-Skornickova JLS1871* (holotype, SING, isotype, M: M0225616)	Vietnam	solitary	rugose	rugose	high	
*A.marasmioides*	BGMN	96/3118; *Arnautov 88-117*	*Tillich 4458* (holotype, M: M0213552)	Vietnam	solitary	rugose	rugose	high	
	MBG	2015.11354.01	*Vislobokov 13046* (MW: MW0735052)	Vietnam	solitary	rugose	rugose	high	

* For description of the abbreviations, see Materials and methods. ** Estimated as number of papillae per 0.01 mm^2^: low = 1–3.5, medium = 4–7.5, high = 8–45.5.
